# Bleeding Colostomy Varices in a Cirrhotic Patient: Successful Management With Glue-Assisted Antegrade and Retrograde Transvenous Obliteration (GAARTO)

**DOI:** 10.14309/crj.0000000000001850

**Published:** 2025-10-01

**Authors:** Ayush Jasrotia, Harshini Revanuru, Pabitra Sahu, Vignesh Kandasamy, Premashis Kar, Sanchit Singh, Jata Shankar Kumar, Subhasish Mazumder, Ashish Garg

**Affiliations:** 1 Department of Medical Gastroenterology, Max Super Speciality Hospitals, Vaishali, Ghaziabad, India; 2 Department of Clinical Sciences, Bridgetown International University, Bridgetown, Barbados; 3 Department of Interventional Radiology, Max Super Speciality Hospitals, Vaishali, Ghaziabad, India

**Keywords:** ectopic varices, transjugular intrahepatic portosystemic shunt, glue-assisted antegrade and retrograde transvenous obliteration, per stoma colonoscopy

## Abstract

Parastomal varices are rare but potentially life-threatening complication of portal hypertension, often presenting with bleeding at stoma sites and frequently underrecognized due to their ectopic location. We report a 76-year-old man with decompensated cirrhosis and metastatic colon cancer who presented with recurrent colostomy site bleeding 14 years after left hemicolectomy. Computed tomography angiography revealed large portosystemic collaterals consistent with parastomal varices. Owing to high risk associated with transjugular intrahepatic portosystemic shunt, he was treated with glue-assisted antegrade and retrograde transvenous obliteration via inferior mesenteric vein access. The procedure achieved effective hemostasis without complications or recurrence. This case highlights the diagnostic value of computed tomography angiography and the therapeutic potential of glue-assisted antegrade and retrograde transvenous obliteration as a less encephalopathy-prone alternative to transjugular intrahepatic portosystemic shunt in high-risk patients.

## INTRODUCTION

Ectopic varices are portosystemic collaterals occurring at sites other than gastroesophageal junction and account for approximately 1%–5% of all variceal bleeding episodes.^1,2^ Among these, parastomal or stomal varices are particularly rare and under-recognized complication of portal hypertension, most often seen in patients who have undergone surgical bowel diversion, such as colostomy or ileostomy.^2,3^ These ectopic varices develop due to increased portal pressure and the formation of collateral vessels between the mesenteric and systemic venous circulations through the surgically created stoma tract.^2,4^

We report a rare case of severe spontaneous bleeding from colostomy varices in a cirrhotic patient with portal hypertension, successfully managed by glue-assisted antegrade and retrograde transvenous obliteration (GAARTO). To our knowledge, such cases are sparsely reported in literature, and this report adds valuable insight into the evolving role of minimally invasive techniques in the management of ectopic variceal hemorrhage.

## CASE REPORT

A 76-year-old man with a history of adenocarcinoma of the colon (status post left hemicolectomy with end colostomy in 2011) presented with spontaneous bleeding from the colostomy site. The patient was a known case of cirrhosis with portal hypertension with previous decompensation of ascites and variceal bleeding. He had biopsy proven liver metastases. The patient was on regular beta-blocker, diuretic, and other supportive therapy. There was no history of any anticoagulant or antiplatelet therapy. There was no history of melena, hematemesis, or local trauma. On examination, the patient was hemodynamically stable but appeared pale. Notable findings included moderate ascites and persistent oozing from the colostomy site without any signs of local infection or inflammation.

Routine investigations showed, Hemoglobin: 8.2 g/dL(dropped to 6.8 g/dL), Platelets: 90,000/mm^3^, creatinine- 1.1, Sodium: 129 mmol/L, Potassium: 3.7 mmol/L, aspartate aminotransferase/alanine aminotransferase- 35.1/17.3 IU/L, Bilirubin Total/Direct Bilirubin-0.5/0.3 mg/dL, alkaline phosphatase/gama glutamyl transferase- 93.3/28.7 IU/L, Albumin- 3.1 g/dL, Ammonia- 73.1 μg/dL. Ultrasound showed cirrhotic liver, splenomegaly, and moderate ascites.

Computed tomography (CT) angiography revealed features of a cirrhotic liver, dilated splenic vein, and large portosystemic collaterals extending through the colostomy site forming a large ectopic parastomal varix.

**Figure 1.**
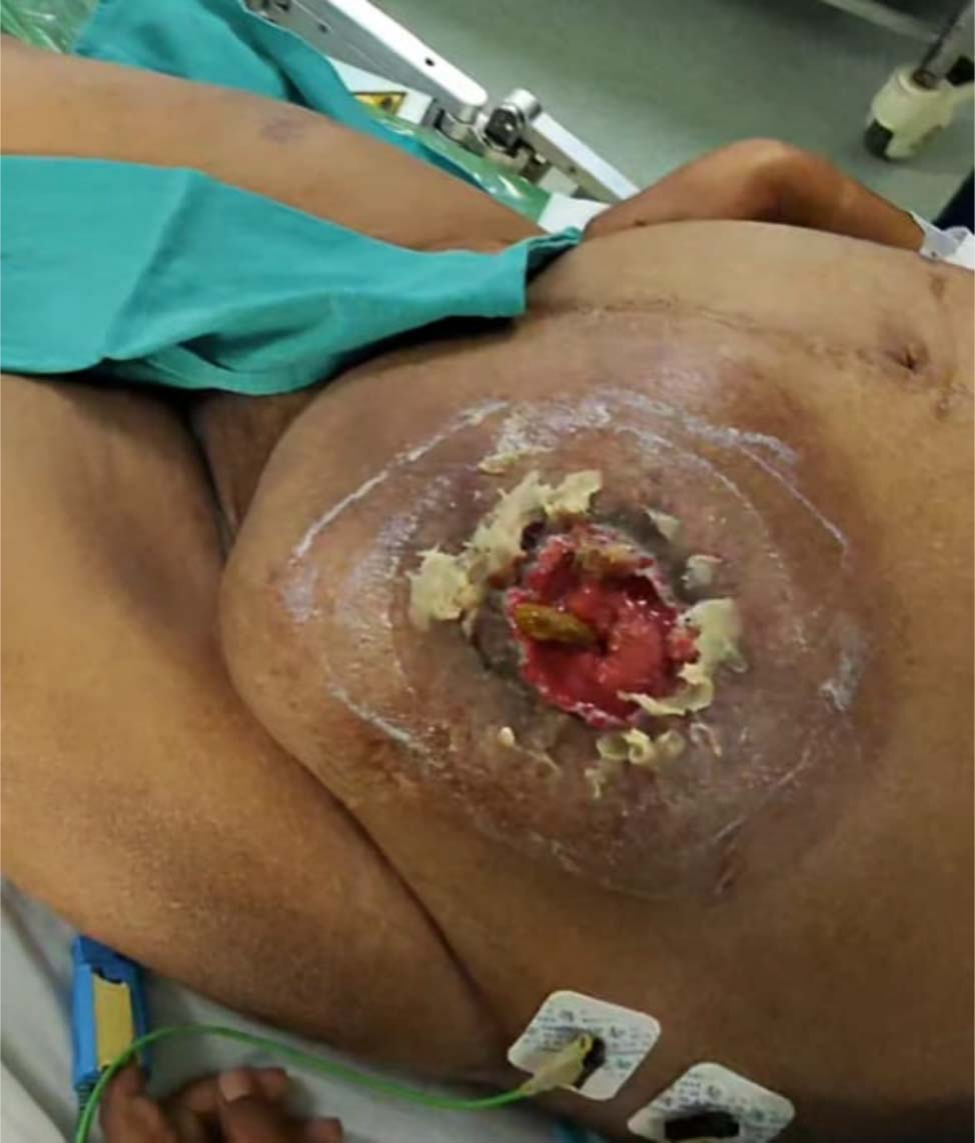
Showing active bleeding from colostomy site with visible ectatic submucosal varices.

On colonoscopy through stomal opening, scope passed up to 50 cm. There was no mucosal varices and no evidence of luminal bleeding. Oozing of blood was seen from area around the stomal opening (Figure 1).

**Figure 2.**
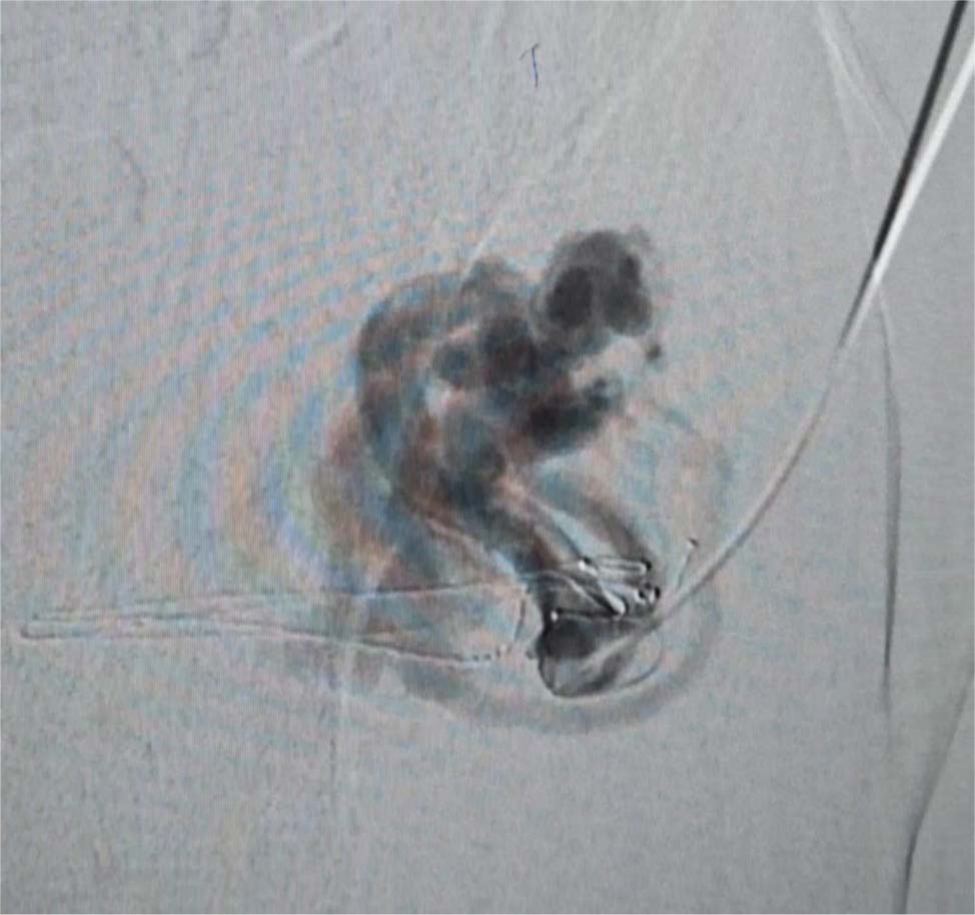
Showing ultra sono graphy-guided variceal feeder puncture and venogram demonstrating the parastomal component of varix and this is followed by ante-grade glue embolization.

**Figure 3.**
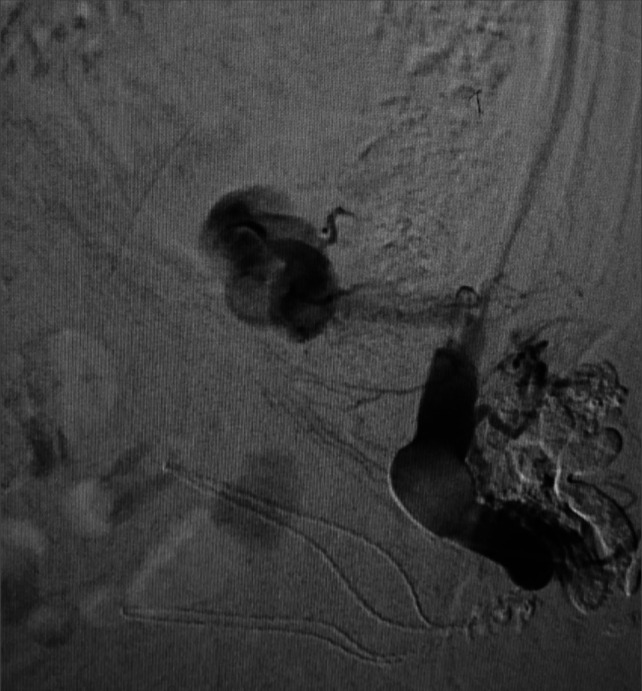
Showing post antegrade glue embolization of parastomal varix and retrograde setrol sclerotherapy for inferior mesenteric vein.

**Figure 4.**
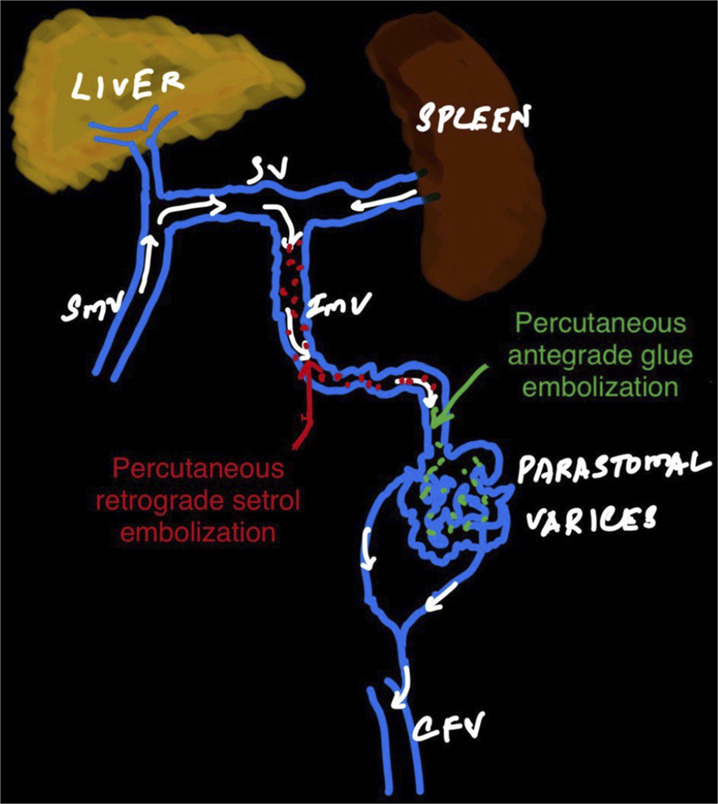
Showing the schematic diagram explaining the location of parastomal varices, the vein supplying the varix and the location where the percutaneous antegrade glue embolization and percutaneous retrograde setrol embolization were performed.

After multidisciplinary discussion, it was decided to perform a GAARTO, which involves direct percutaneous puncture of the superficial part of inferior mesenteric vein supplying the varix followed by Antegrade glue embolization of Parastomal varix and Retrograde setrol sclerotherapy for inferior mesenteric vein (Figures 2-4). Transjugular intrahepatic portosystemic shunt (TIPS) + Coil embolization was not considered in view of metastatic disease, advanced age of patient. Postprocedure, bleeding was resolved and hemoglobin stabilized. There was no rebleeding on follow-up.

## DISCUSSION

Bleeding stomal varices represent a rare and challenging manifestation of portal hypertension.

CT angiography or CT portography revealing ectopic varices, alongside direct stomal endoscopy, is diagnostic.^2,4,5^ In our case, CT angiography confirmed a large parastomal variceal collateral, but colonoscopy through stomal opening did not reveal any varices or bleeding source.

Pharmacologic therapy like non-selective beta-blockers and vasoactive agents (terlipressin, somatostatin analogues) may reduce portal pressure, but are often insufficient for stomal varices.^3,6^ In our case, the patient was given terlipressin infusion along with tranexamic acid and vitamin K as supportive measures.

TIPS with coil embolization is widely recognized as first-line for refractory stomal variceal bleeding. Transvenous obliteration procedures—including balloon-occluded retrograde transvenous obliteration (BRTO), plug-assisted retrograde transvenous obliteration, coil-assisted retrograde transvenous obliteration, balloon-assisted antegrade transvenous obliteration, and GAARTO—have gained popularity for their efficacy in gastric varices and are now extrapolated to stomal varices.^2,3,7-9^ Although BRTO is well-described for gastric varices,^7,10^ GAARTO provides enhanced obliteration through dual antegrade and retrograde access, thus causing complete obliteration of varix, preventing future recanalization. A recent case series demonstrated successful coil embolization in stomal varices, with postprocedure imaging confirming complete occlusion and no recurrence.^11^ GAARTO as used in our case, mirrors the benefits of combined antegrade and retrograde embolization seen in gastric varices.^3^ This technique ensures targeted treatment of afferent feeding veins and confirmation of thrombosis via systemic backflow. Compared with TIPS, GAARTO and similar techniques avoid risk of hepatic encephalopathy as they target isolated variceal beds without altering systemic and portal hemodynamics.^2,7,10^

Long-term studies in gastric varices report low rebleeding rates (≤10%) and improved hepatic hemodynamics after BRTO.^7^ Though data on stomal varices are limited, selective embolization or GAARTO has shown promise in maintaining durable hemostasis without major complications.^6,11^ As in our patient, GAARTO can be a very effective alternative to TIPS in high-risk patients with ectopic stomal variceal bleed and carries lesser risk of hepatic encephalopathy.

Bleeding from parastomal varices is a rare but serious complication of portal hypertension that presents unique diagnostic and therapeutic challenges. Although traditional options such as TIPS and coil embolization remain standard approaches, minimally invasive transvenous obliteration techniques are emerging as effective alternatives, particularly in high-risk patients. GAARTO offers a distinct advantage by achieving durable variceal obliteration without significantly altering portal-systemic hemodynamics. Unlike TIPS, which is associated with an increased risk of hepatic encephalopathy due to diversion of portal flow, GAARTO provides targeted embolization of afferent and efferent veins while preserving physiologic portal circulation. Our case highlights the successful use of GAARTO in achieving durable hemostasis without recurrence. This experience underscores the evolving role of targeted endovascular interventions in the management of ectopic variceal bleeding and supports GAARTO as a valuable option in patients who are elderly, have advanced comorbidities where conventional therapies may be unsuitable.

## DISCLOSURES

Author contributions: A. Jasrotia and H. Revanuru were involved in drafting the original manuscript, data collection, literature review. P. Kar, P. Sahu, and S. Singh were involved in diagnosing, managing the patient, and resourcing, and reviewing the manuscript, V. Kandasamy was involved in Radiological diagnosis and Intervention, drafting and reviewing the manuscript, JS Kumar, S. Mazumder, A. Garg, were involved in reviewing and editing the manuscript. P. Kar is the article guarantor.

Financial disclosure: None to report.

Informed consent was obtained for this case report.

## ABBREVIATIONS:

TIPS: Transjugular Intrahepatic Portosystemic Shunt
EVL: Endoscopic Variceal Ligation
GAARTO: Glue-Assisted Antegrade and Retrograde Transvenous Obliteration
BRTO: Balloon-Occluded Retrograde Transvenous Obliteration
PARTO: Plug-Assisted Retrograde Transvenous Obliteration
CARTO: Coil-Assisted Retrograde Transvenous Obliteration
BATO: Balloon-Assisted Antegrade Transvenous Obliteration
